# Supplementary material for: A comprehensive benchmarking with interpretation and operational guidance for the hierarchy of topologically associating domains
Source: Nat Commun. 2024 May 23;15:4376. doi: 10.1038/s41467-024-48593-7 (PMC11116433; doi:10.1038/s41467-024-48593-7)
Supplement: Supplementary file 1 — Supplementary Information [file 41467_2024_48593_MOESM1_ESM.pdf]

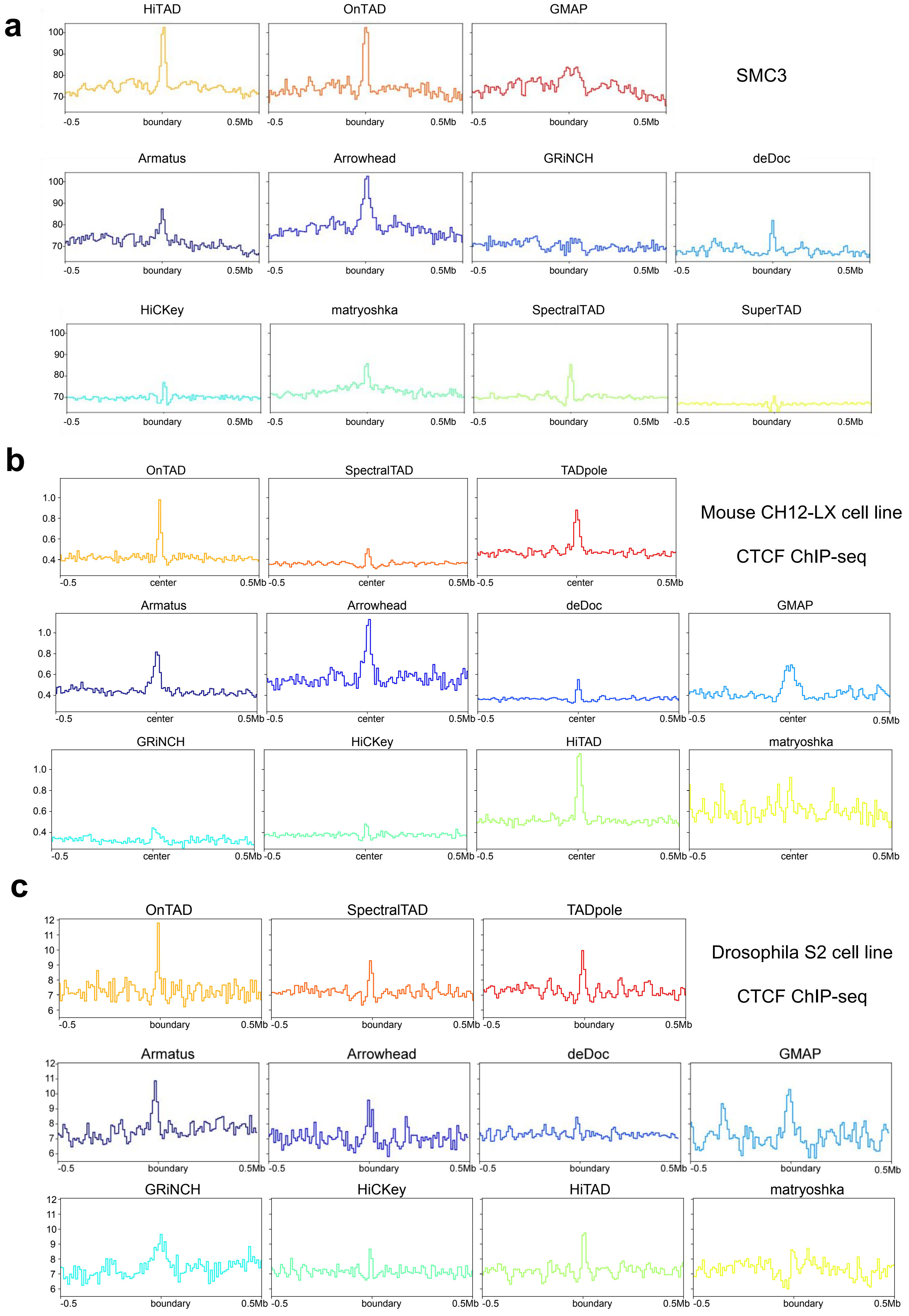

a
HiTAD
OnTAD
GMAP
SMC3
Armatus
Arrowhead
GRiNCH
deDoc
HiCKey
matryoshka
SpectralTAD
SuperTAD
100
90
80
70
-0.5
boundary
0.5Mb
b
Mouse CH12-LX cell line
CTCF ChIP-seq
OnTAD
SpectralTAD
TADpole
Armatus
Arrowhead
deDoc
GMAP
GRiNCH
HiCKey
HiTAD
matryoshka
1.0
0.8
0.6
0.4
-0.5
center
0.5Mb
c
Drosophila S2 cell line
CTCF ChIP-seq
OnTAD
SpectralTAD
TADpole
Armatus
Arrowhead
deDoc
GMAP
GRiNCH
HiCKey
HiTAD
matryoshka
12
11
10
9
8
7
6
-0.5
boundary
0.5Mb

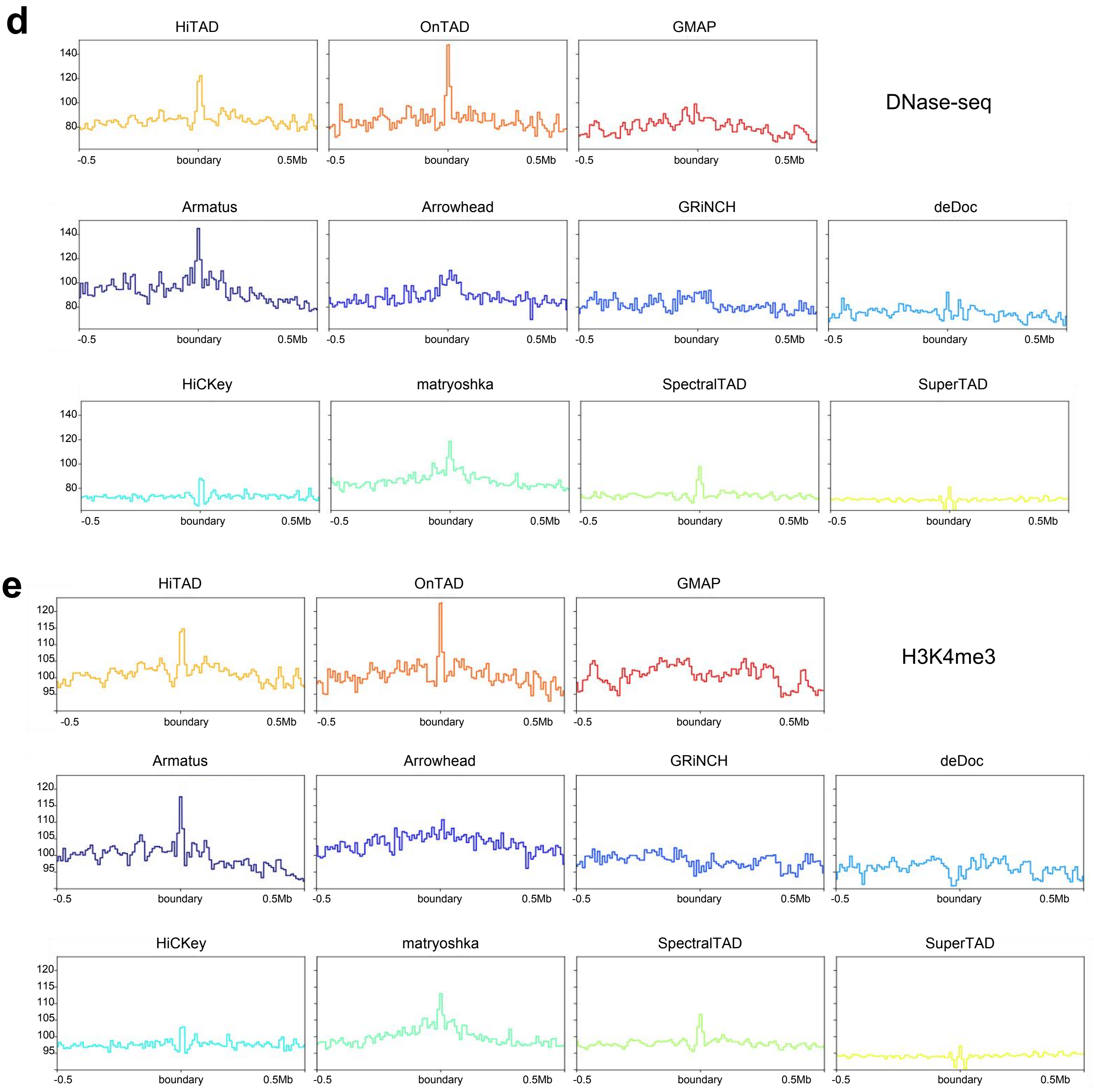

Supplementary Figure 1. Representative examples of peak signals for (a) SMC3 (b) CTCF in mouse CH12-LX cell line (c) CTCF in drosophila S2 cell line (d) DNase-seq and (e) H3K4me3.

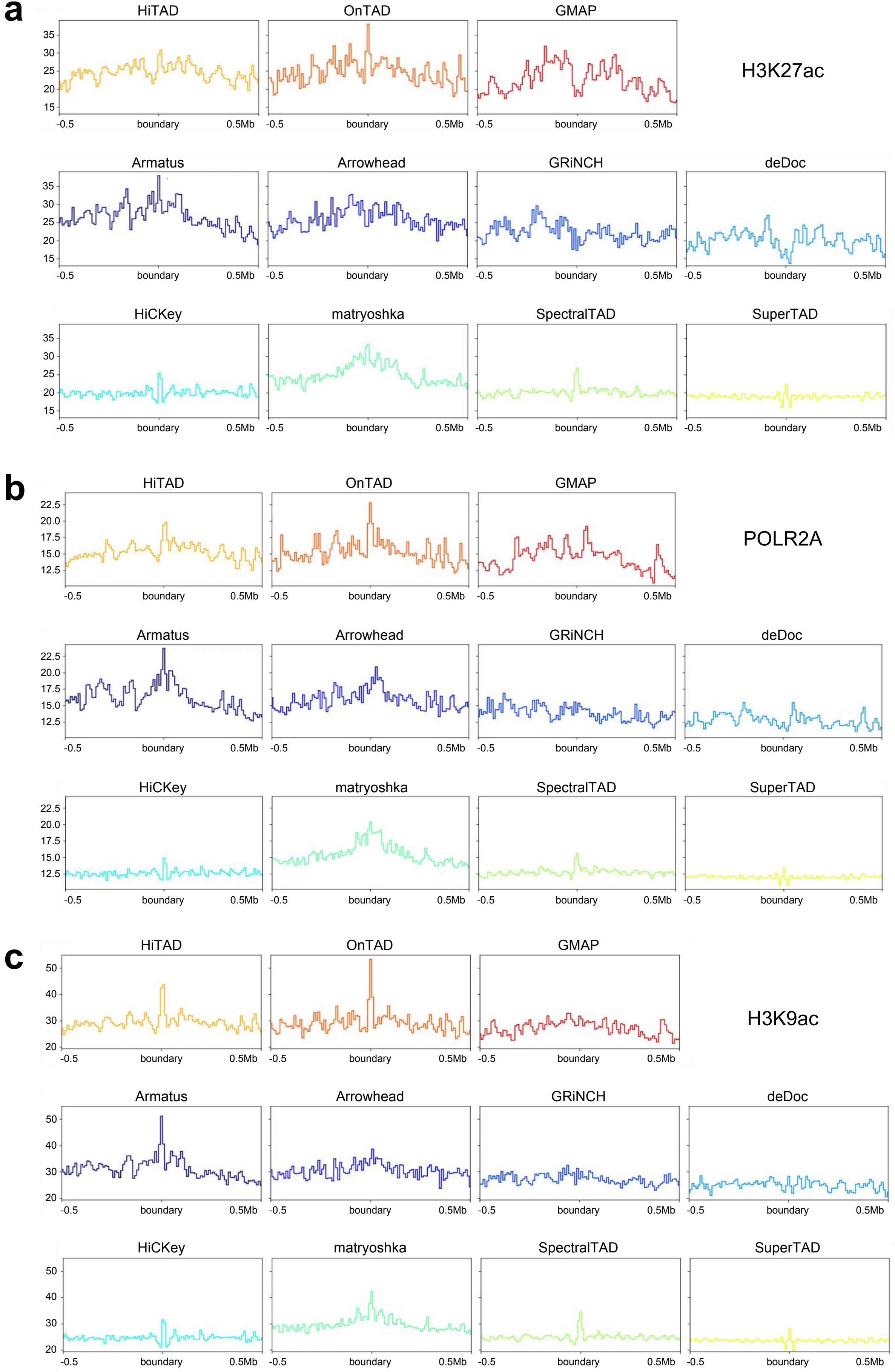

Supplementary Figure 2. Representative examples of ChIP-seq peak signals for (a) H3K27ac (b) POLR2A and (c) H3K9ac.

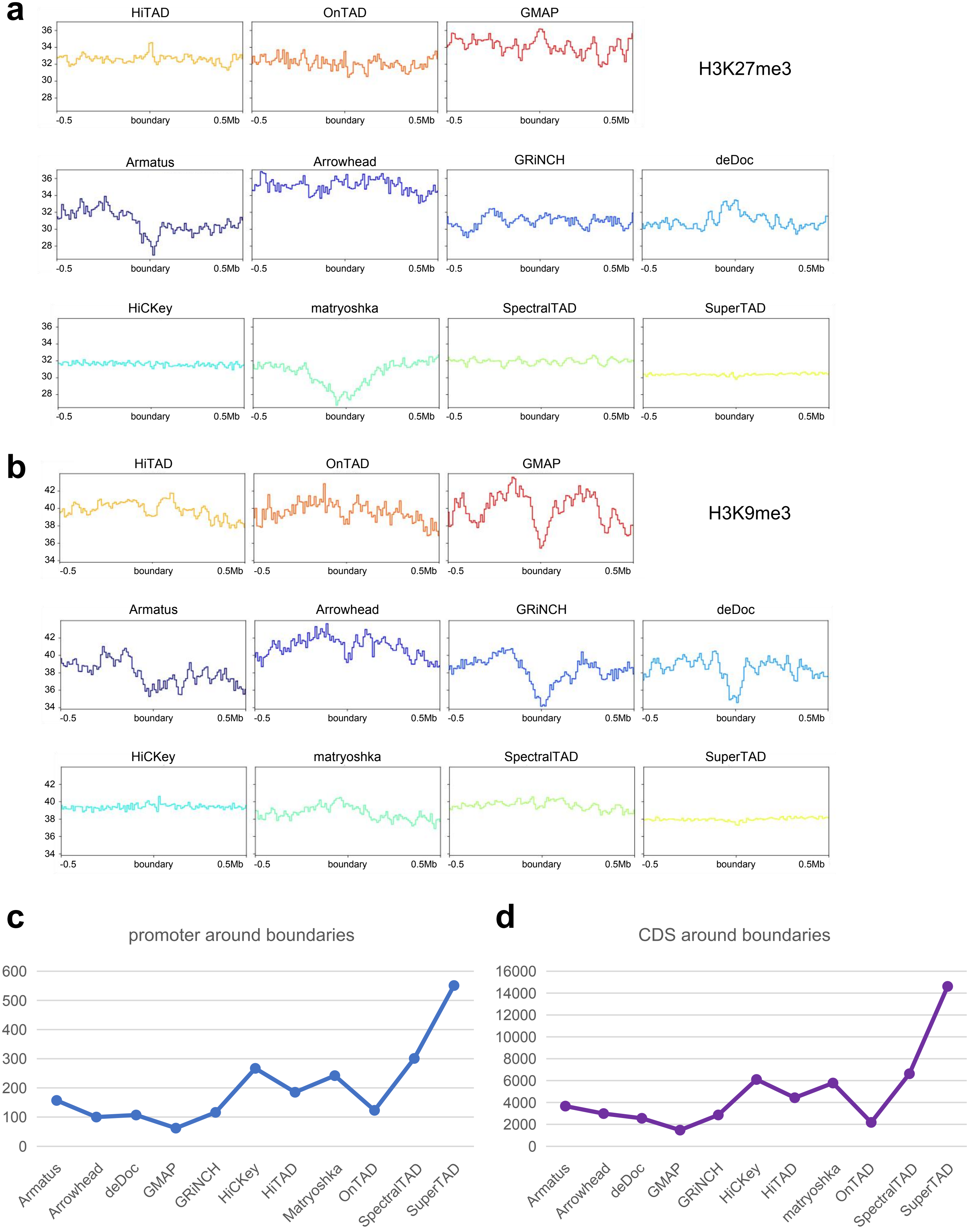

Supplementary Figure 3. Representative examples of ChIP-seq peak signals for (a) H3K27me3 (b) H3K9me3. (c) Numbers of overlap between TAD boundaries and (c) promoter or (d) CDS

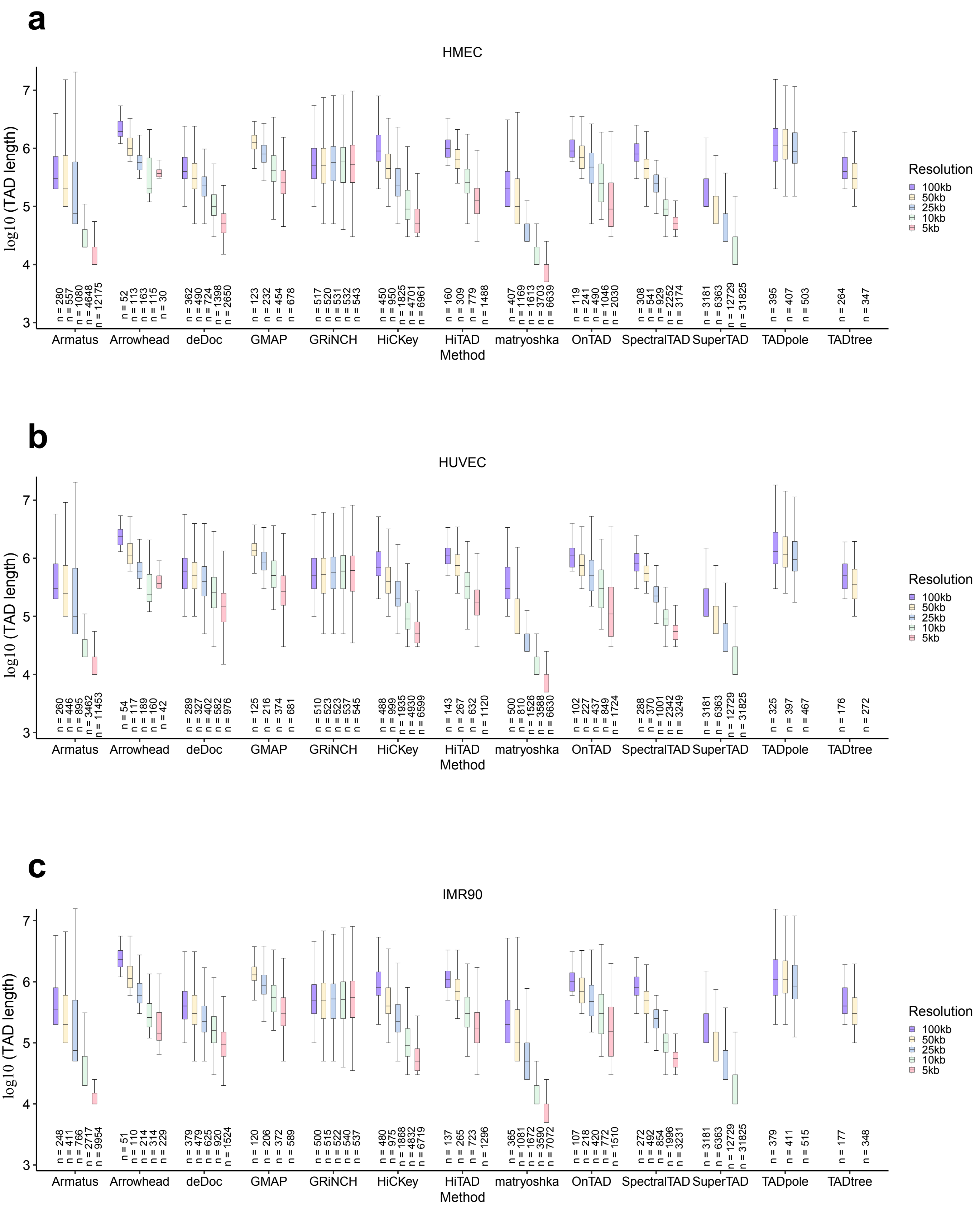
a
HMEC
log10 (TAD length)
n = 280 n = 557 n = 1080 n = 4648 n = 12175
n = 52 n = 113 n = 163 n = 115 n = 30
n = 362 n = 490 n = 724 n = 1398 n = 2650
n = 123 n = 232 n = 454 n = 678
n = 517 n = 520 n = 531 n = 532 n = 543
n = 450 n = 950 n = 1825 n = 4701 n = 6961
n = 160 n = 309 n = 779 n = 1488
n = 407 n = 1169 n = 1613 n = 3703 n = 6639
n = 119 n = 241 n = 490 n = 1046 n = 2030
n = 308 n = 541 n = 929 n = 2252 n = 3174
n = 3181 n = 6363 n = 12729 n = 31825
n = 395 n = 407 n = 503
n = 264 n = 347
Armatus Arrowhead deDoc GMAP GRiNCH HiCKey HiTAD matryoshka OnTAD SpectralTAD SuperTAD TADpole TADtree
Method
Resolution
100kb
50kb
25kb
10kb
5kb
b
HUVEC
log10 (TAD length)
n = 260 n = 446 n = 895 n = 3462 n = 11453
n = 54 n = 117 n = 189 n = 160 n = 42
n = 289 n = 327 n = 402 n = 582 n = 976
n = 125 n = 216 n = 374 n = 681
n = 510 n = 523 n = 523 n = 537 n = 545
n = 488 n = 999 n = 1935 n = 4930 n = 6599
n = 143 n = 267 n = 632 n = 1120
n = 500 n = 810 n = 1526 n = 3588 n = 6630
n = 102 n = 227 n = 437 n = 849 n = 1724
n = 288 n = 370 n = 1001 n = 2342 n = 3249
n = 3181 n = 6363 n = 12729 n = 31825
n = 325 n = 397 n = 467
n = 176 n = 272
Armatus Arrowhead deDoc GMAP GRiNCH HiCKey HiTAD matryoshka OnTAD SpectralTAD SuperTAD TADpole TADtree
Method
Resolution
100kb
50kb
25kb
10kb
5kb
c
IMR90
log10 (TAD length)
n = 248 n = 411 n = 766 n = 2717 n = 9954
n = 51 n = 110 n = 214 n = 314 n = 229
n = 379 n = 479 n = 625 n = 920 n = 1524
n = 120 n = 206 n = 372 n = 589
n = 500 n = 515 n = 522 n = 540 n = 537
n = 480 n = 975 n = 1868 n = 4832 n = 6719
n = 137 n = 265 n = 723 n = 1296
n = 365 n = 1081 n = 1672 n = 3590 n = 7072
n = 107 n = 218 n = 420 n = 772 n = 1510
n = 272 n = 492 n = 854 n = 1996 n = 3231
n = 3181 n = 6363 n = 12729 n = 31825
n = 379 n = 411 n = 515
n = 177 n = 348
Armatus Arrowhead deDoc GMAP GRiNCH HiCKey HiTAD matryoshka OnTAD SpectralTAD SuperTAD TADpole TADtree
Method
Resolution
100kb
50kb
25kb
10kb
5kb

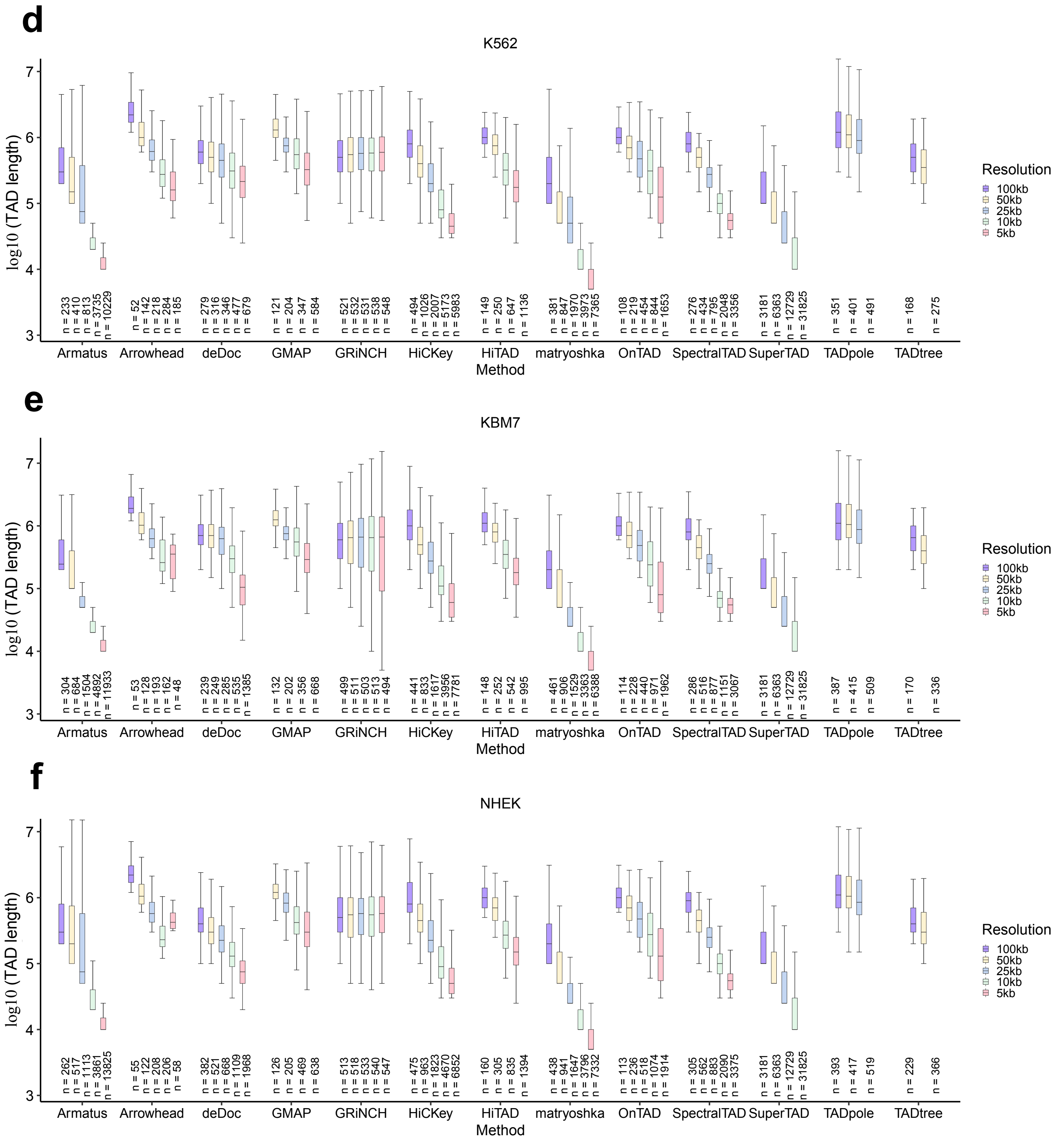

Supplementary Figure 4. TAD size range at various resolutions in chromosome 7 of ICE-normalized Hi-C data. The line that divides the box into 2 parts represents the median of the data. The ends of the box shows the upper (Q3) and lower (Q1) quartiles. The difference between Quartiles 1 and 3 is called the interquartile range (IQR). The extreme line shows Q3+1.5xIQR to Q1-1.5xIQR (the highest and lowest value excluding outliers). These graphs are from the following cell lines: (a) HMEC, (b) HUVEC, (c) IMR90, (d) K562, (e) KBM7, and (f) NHEK. Source data are provided as a Source Data file.

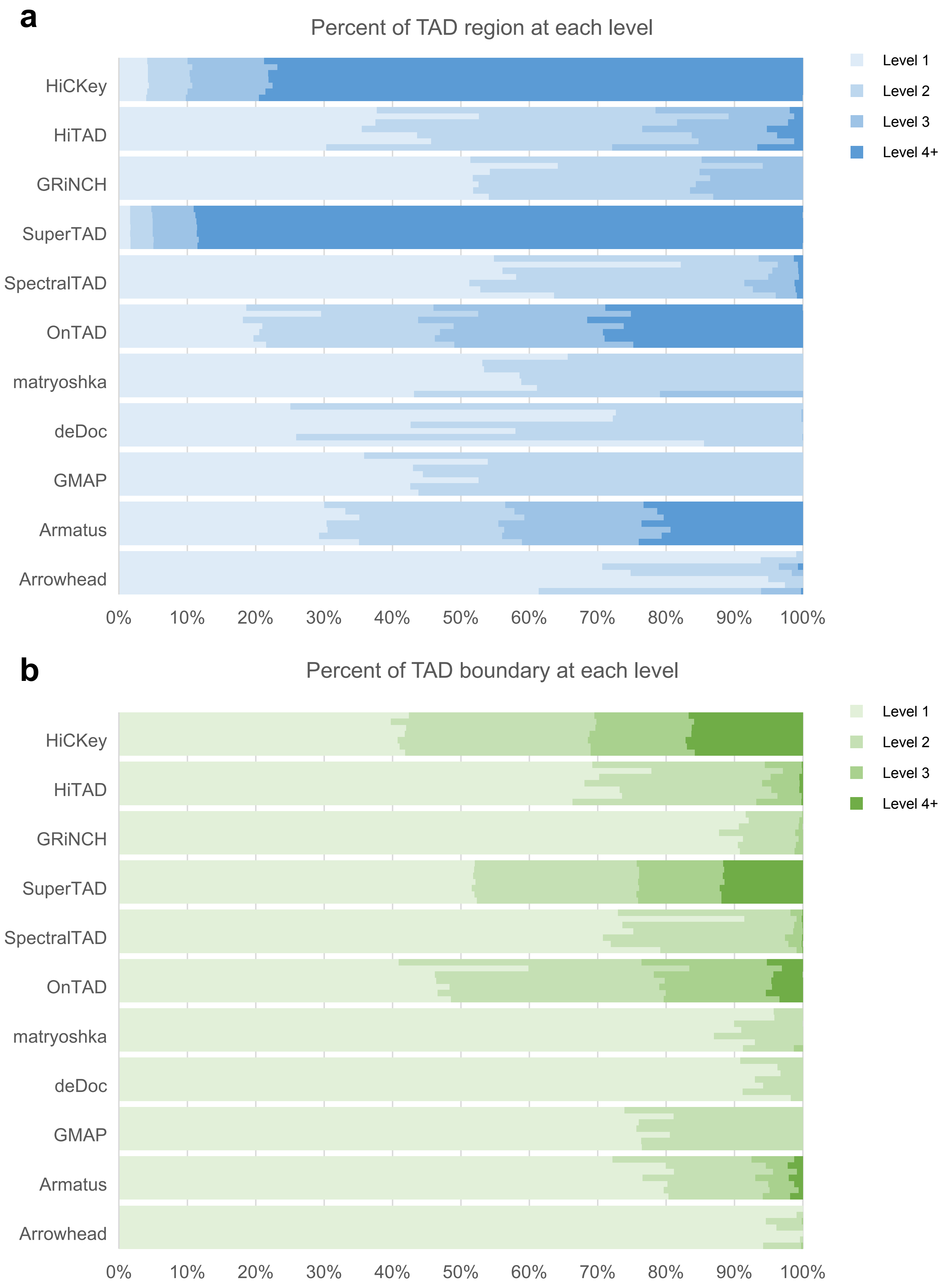
a
Percent of TAD region at each level
HiCKey
HiTAD
GRiNCH
SuperTAD
SpectralTAD
OnTAD
matryoshka
deDoc
GMAP
Armatus
Arrowhead
0%
10%
20%
30%
40%
50%
60%
70%
80%
90%
100%
Level 1
Level 2
Level 3
Level 4+
b
Percent of TAD boundary at each level
HiCKey
HiTAD
GRiNCH
SuperTAD
SpectralTAD
OnTAD
matryoshka
deDoc
GMAP
Armatus
Arrowhead
0%
10%
20%
30%
40%
50%
60%
70%
80%
90%
100%
Level 1
Level 2
Level 3
Level 4+

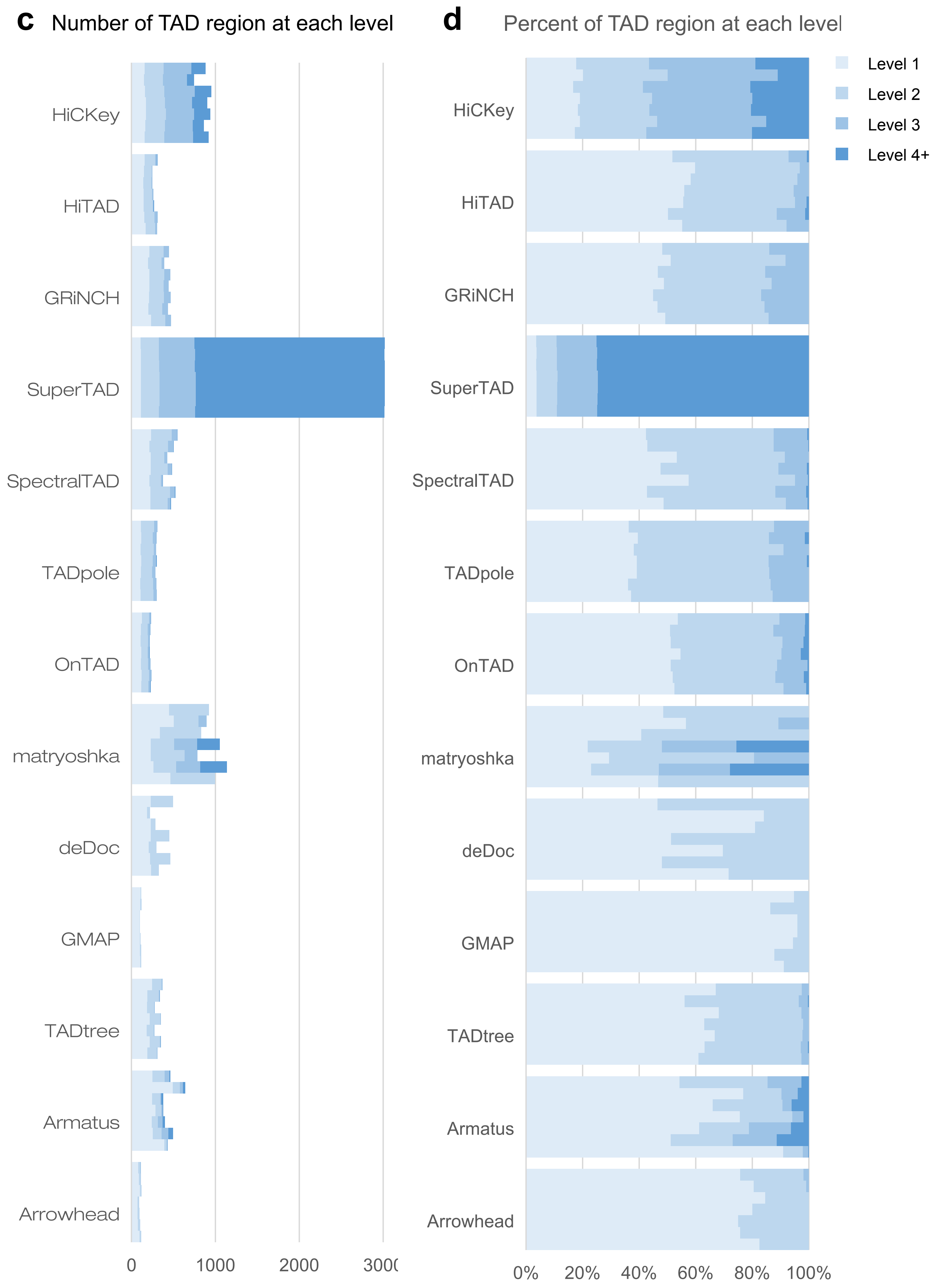
c
Number of TAD region at each level
HiCKey
HiTAD
GRiNCH
SuperTAD
SpectralTAD
TADpole
OnTAD
matryoshka
deDoc
GMAP
TADtree
Armatus
Arrowhead
0
1000
2000
d
Percent of TAD region at each level
HiCKey
HiTAD
GRiNCH
SuperTAD
SpectralTAD
TADpole
OnTAD
matryoshka
deDoc
GMAP
TADtree
Armatus
Arrowhead
0%
20%
40%
60%
80%
100%
Level 1
Level 2
Level 3
Level 4+

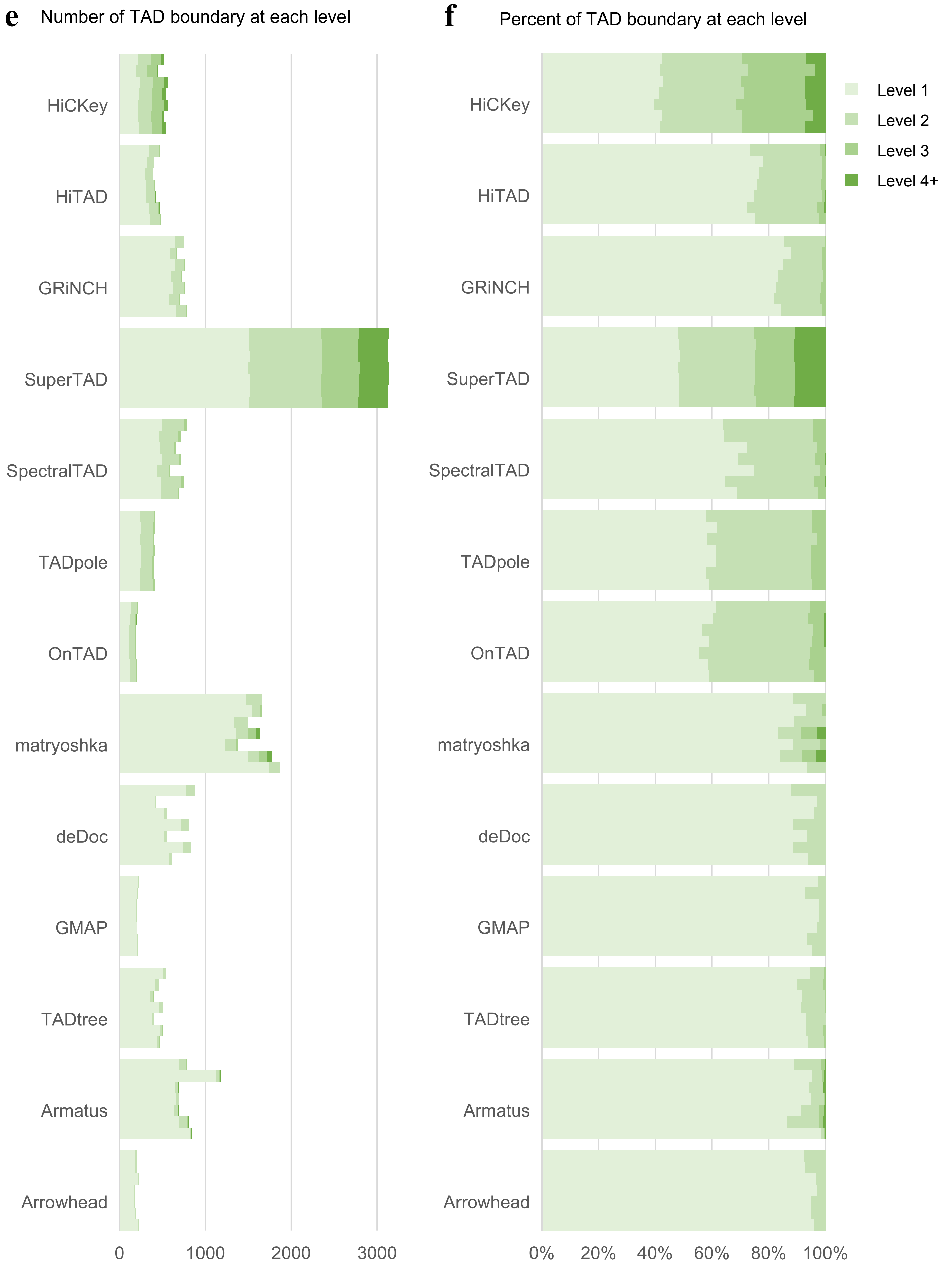
e
Number of TAD boundary at each level
f
Percent of TAD boundary at each level
Level 1
Level 2
Level 3
Level 4+
HiCKey
HiTAD
GRiNCH
SuperTAD
SpectralTAD
TADpole
OnTAD
matryoshka
deDoc
GMAP
TADtree
Armatus
Arrowhead
0
1000
2000
3000
0%
20%
40%
60%
80%
100%

Supplementary Figure 5. Hierarchical TADs in chromosome 7 of GM12878 from ICE-normalized Hi-C data. (a) Ratios of TADs (a) and boundaries (b) at various levels of the GM12878 cell line on 10Kb. (c, d) Numbers (c) and ratios (d) of TADs at various levels of GM12878 cell line on 50Kb. (e, f) Numbers (e) and ratios (f) of boundaries at various levels of GM12878 cell line on 50Kb. Source data are provided as a Source Data file.

a

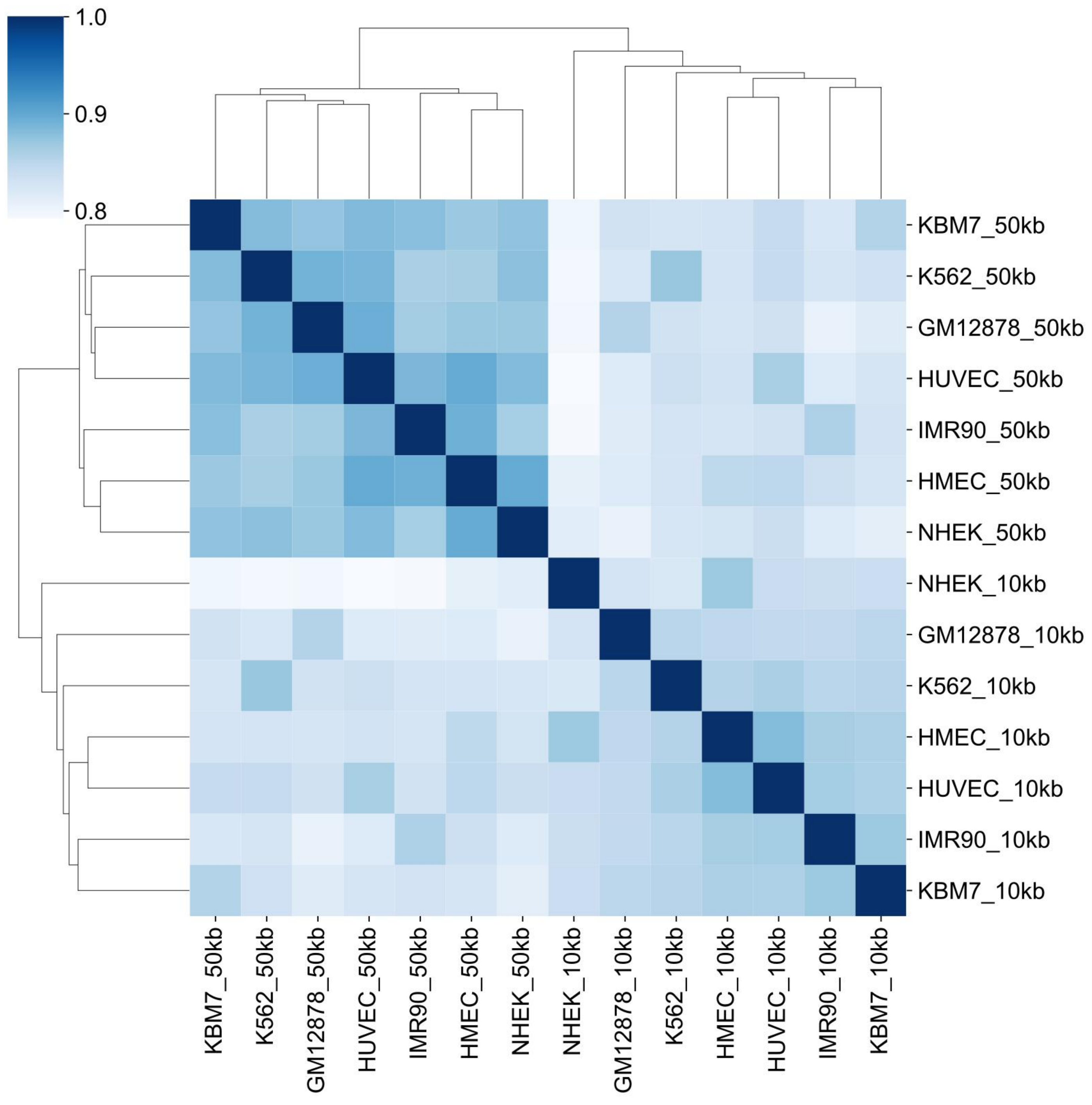

1.0
0.9
0.8
KBM7_50kb
K562_50kb
GM12878_50kb
HUVEC_50kb
IMR90_50kb
HMEC_50kb
NHEK_50kb
NHEK_10kb
GM12878_10kb
K562_10kb
HMEC_10kb
HUVEC_10kb
IMR90_10kb
KBM7_10kb

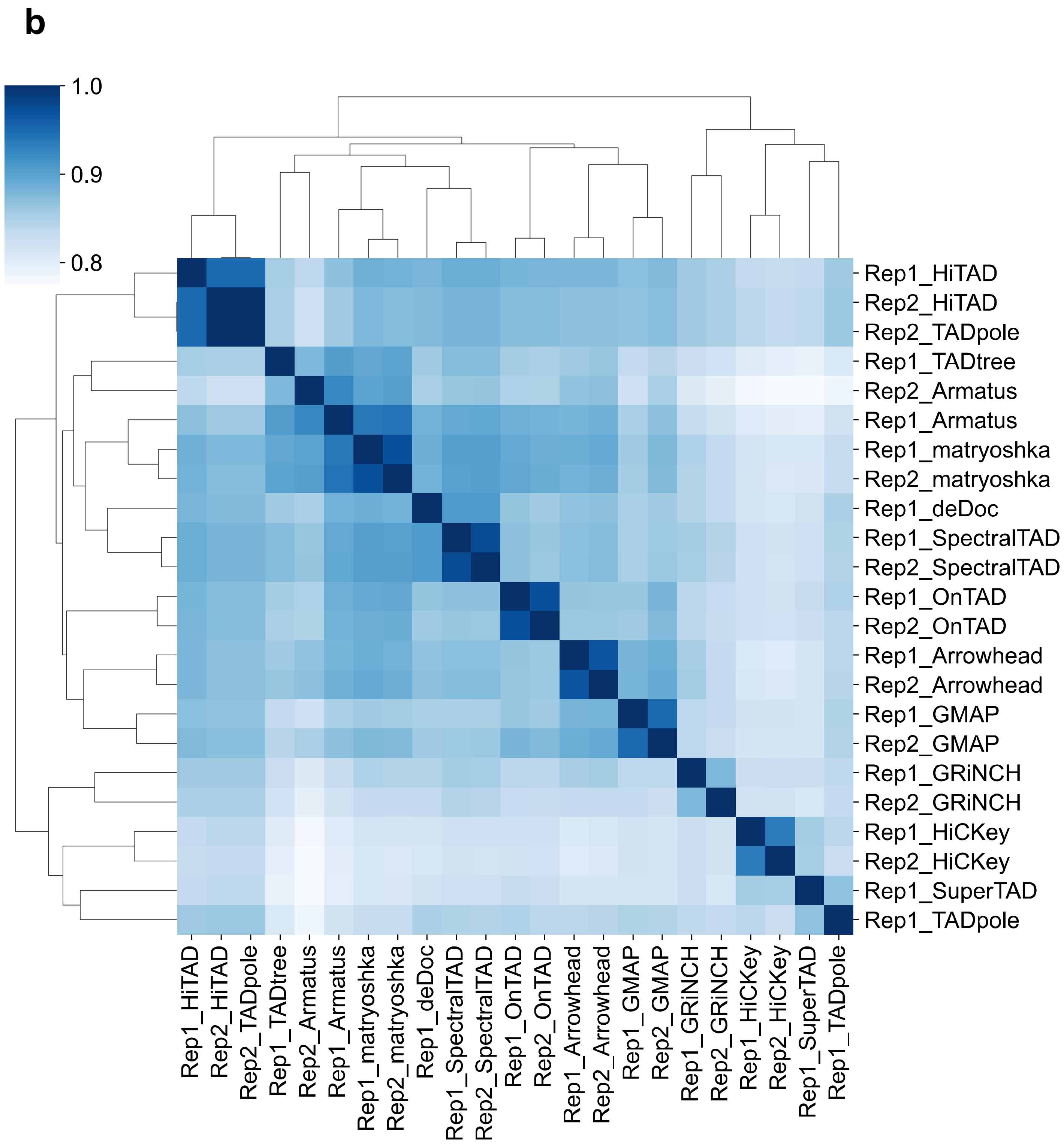

Supplementary Figure 6. Hier_SSIM among different resolutions, cell lines and biological replicates. (a) Hier_SSIM among TAD hierarchy obtained with 7 cell lines on 50Kb ICE data by OnTAD. (b) Hier_SSIM among biological replicates in GM12878. Source data are provided as a Source Data file.

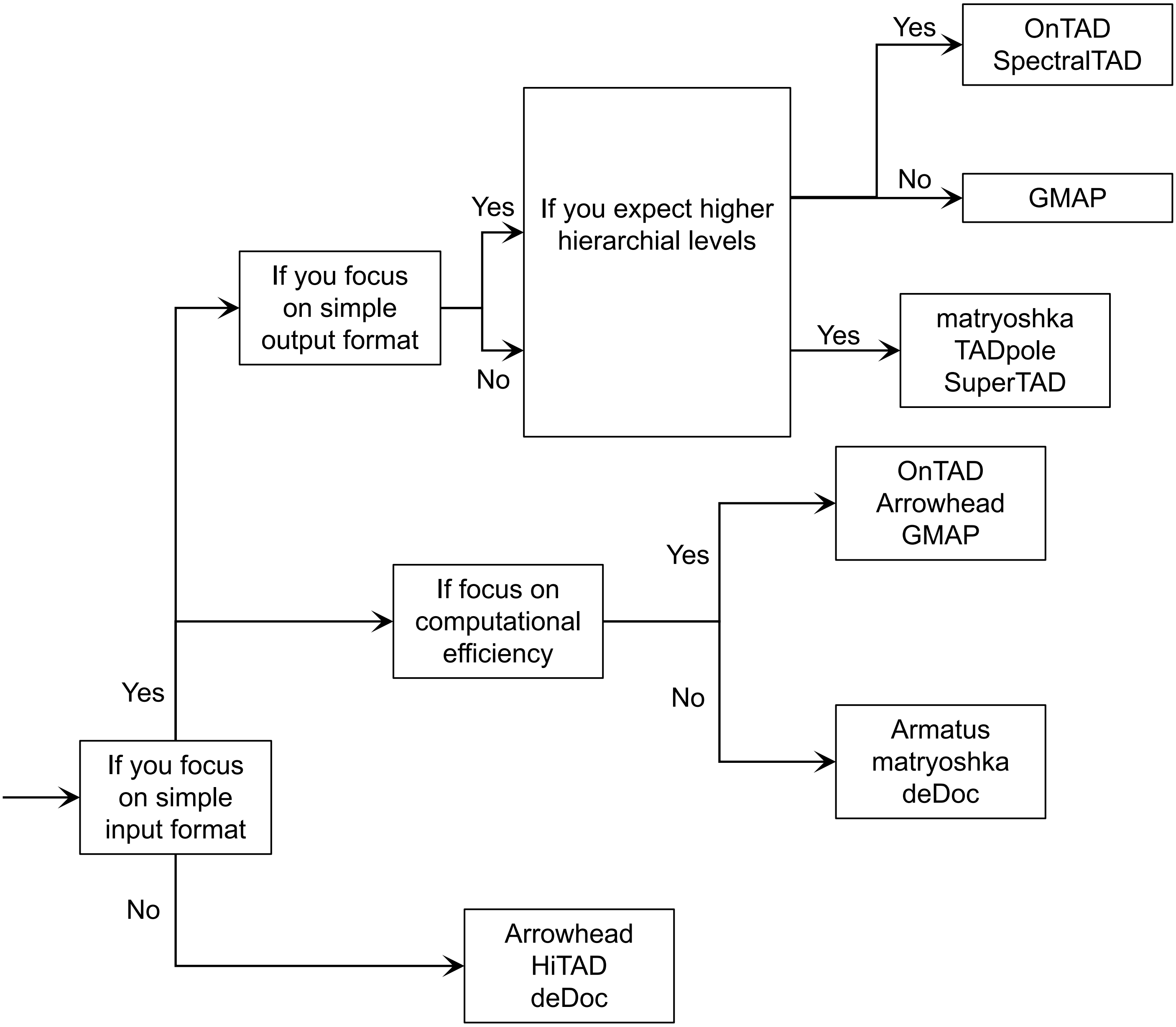

Supplementary Figure 7. User-guidance for TAD hierarchy callers.

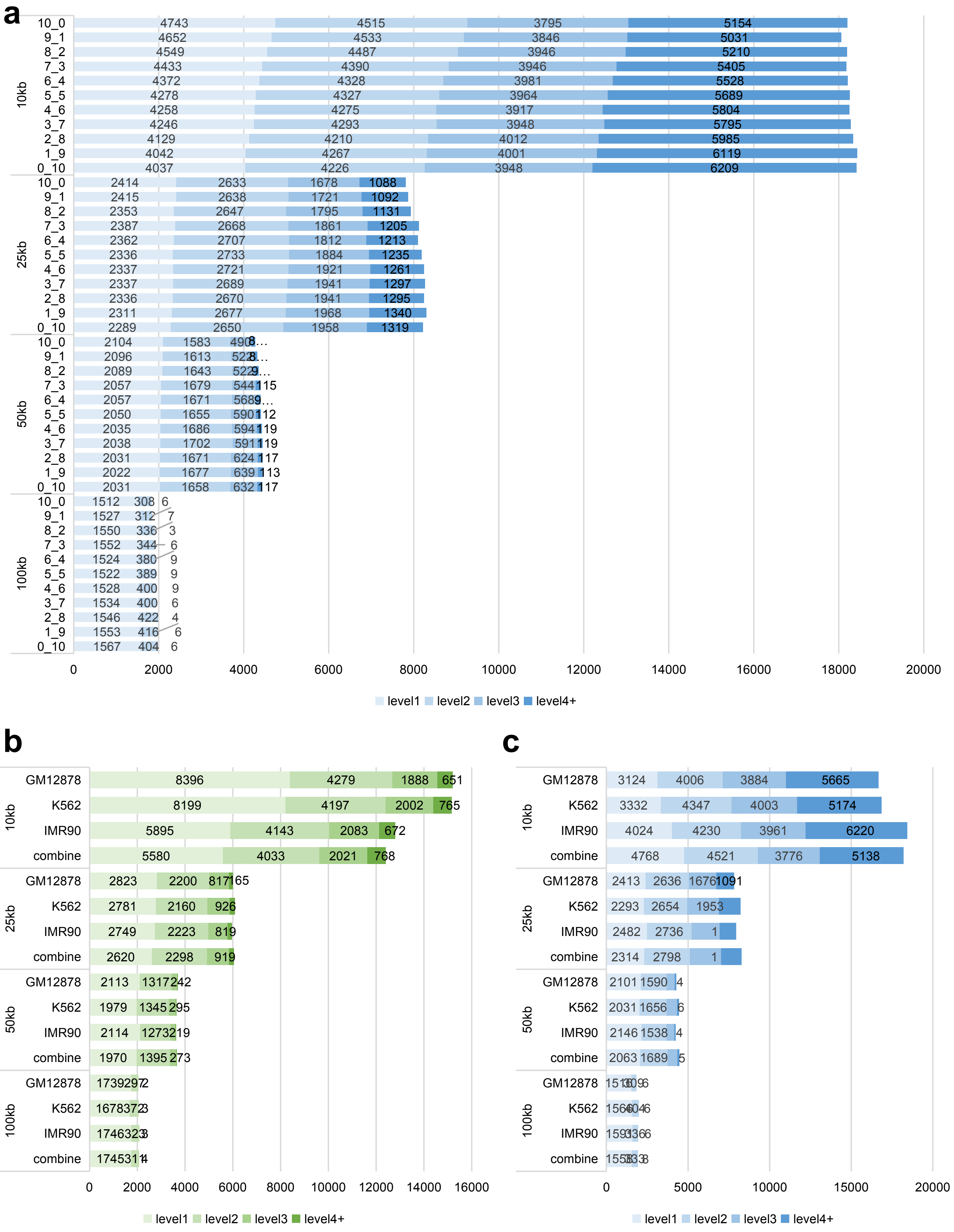
a
b
c
10kb
25kb
50kb
100kb
level1
level2
level3
level4+
GM12878
K562
IMR90
combine

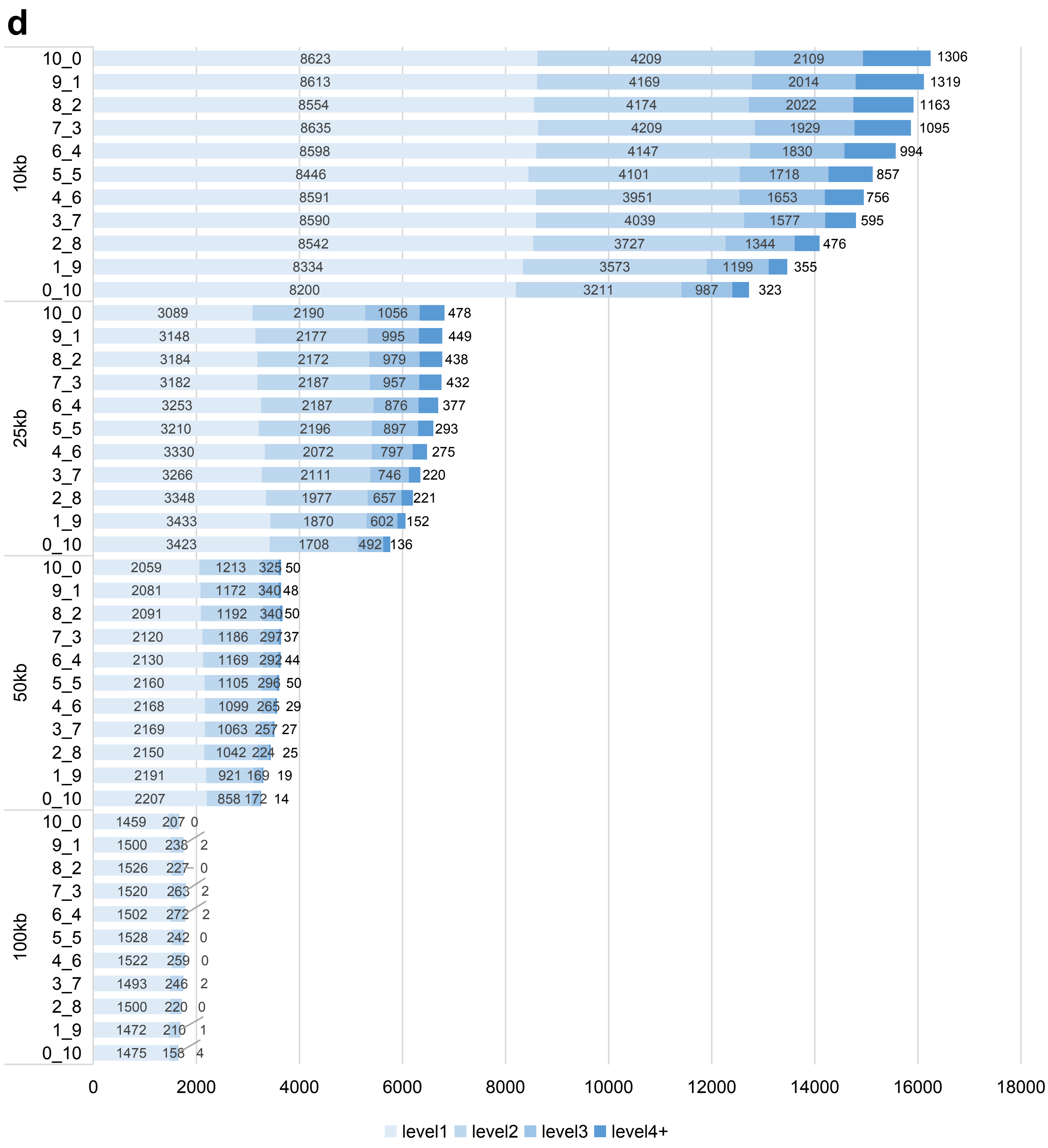

Supplementary Figure 8. TAD hierarchy of mixed samples.
(a) Number of TADs at seperate levels and various resolutions in mixed samples. (b, c) Number of boundaries (b) and TADs (c) at seperate levels and various resolutions in GM12878 cell, K562 cell, IMR90 cell and mixed samples (from top to bottom). (d) Number of TADs at seperate levels and various resolutions in Hi-C of mixed single cell samples.

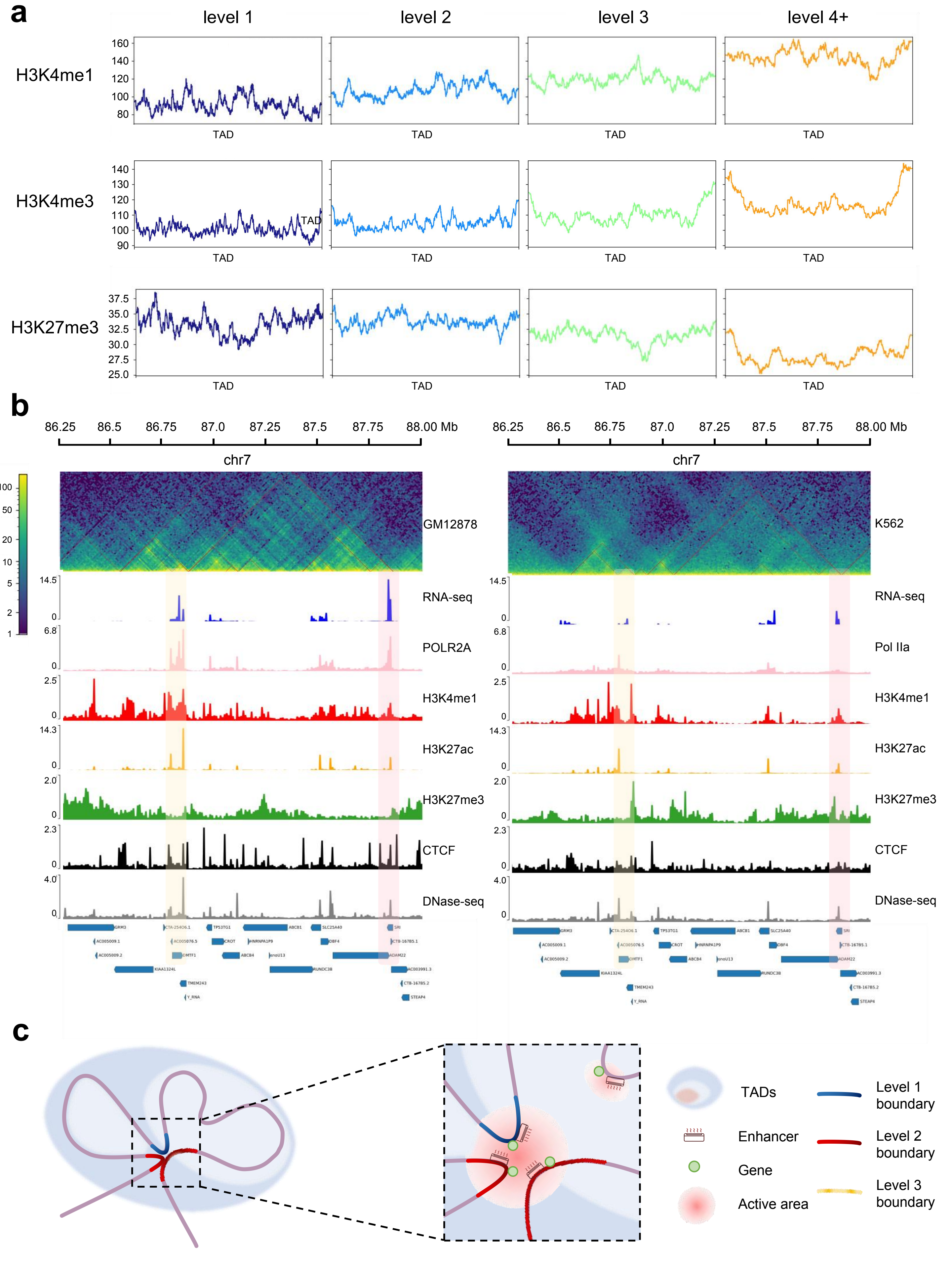
a
level 1
level 2
level 3
level 4+
H3K4me1
H3K4me3
H3K27me3
TAD
b
86.25
86.5
86.75
87.0
87.25
87.5
87.75
88.00 Mb
chr7
GM12878
K562
RNA-seq
POLR2A
Pol IIa
H3K4me1
H3K27ac
H3K27me3
CTCF
DNase-seq
c
TADs
Enhancer
Gene
Active area
Level 1 boundary
Level 2 boundary
Level 3 boundary

Supplementary Figure 9. Histone modifications within TADs of all levels.
(a) Enrichment of H3K4me1 (upper panel), H3K4me3 (middle panel), and H3K27me3 (lower panel) in TADs of level 1, 2, 3, and 4 plus.
(b) Representative example of TAD hierarchy and multiomics landscape in GM12878 cell (left panel) and K562 cell (right panel). The upper panels in both sides show distribution of Hi-C data. Areas with yellow and pink shading are selected to depict inter-cellular variation. (c) Schematic representation of the air conditioner model for TAD hierarchy.

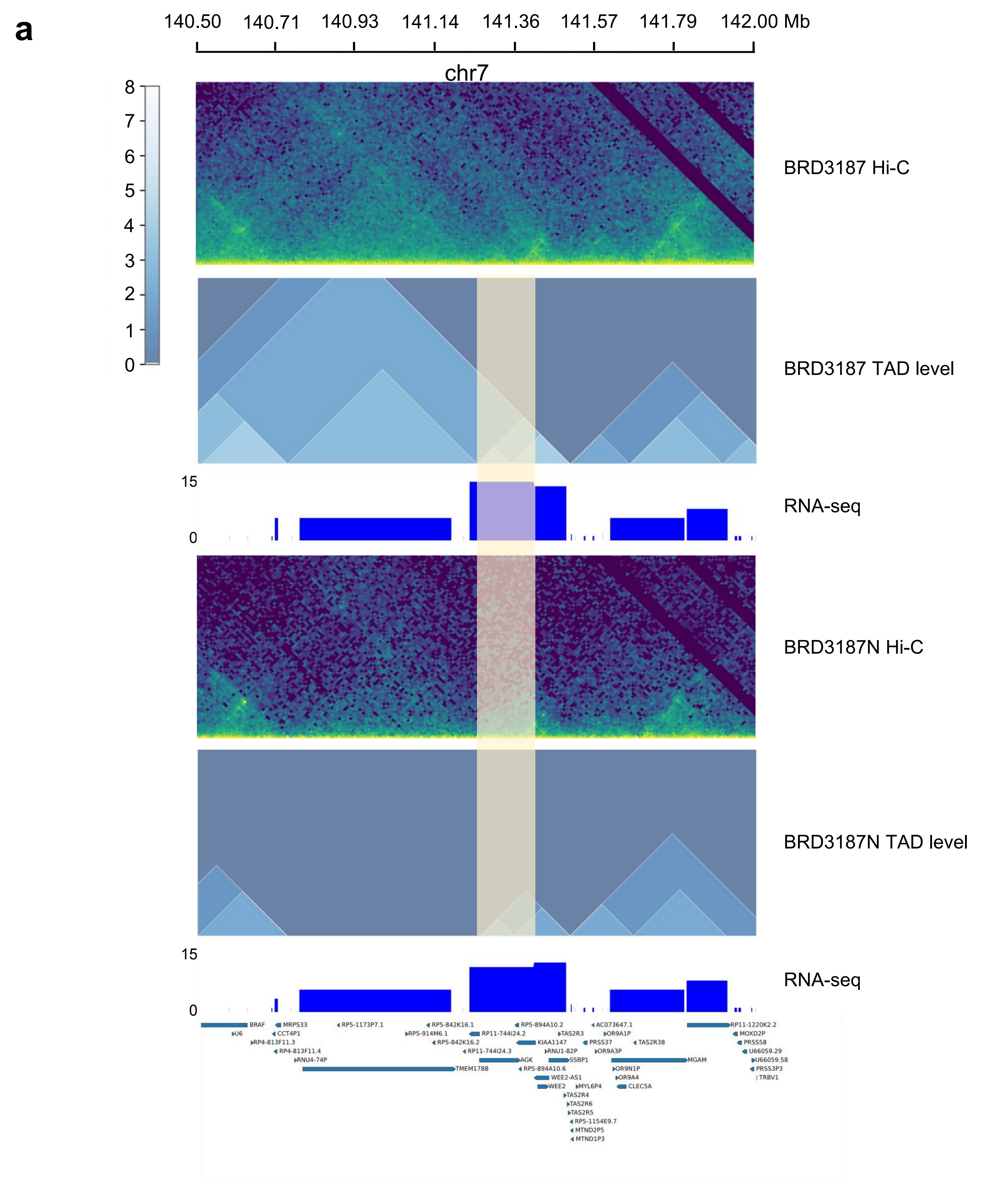

Supplementary Figure 10. TAD hierarchy in colorectal cacinoma and paracancerous tissue. (a) Representative example of TAD hierarchy and multiomics landscape in colorectal carcinoma (BRD3187, upper panel) and that healthy paracancerous tissue (BRD3187N, lower panel). The green images show Hi-C heatmaps in both sides, and the blue images show distribution of TAD hierarchy. Yellow shading shows *AKG* TAD.

Supplementary Table 1

Overlap raio between TAD hierarchy obtained at different resolutions in a pairwise measurement

	5kb-10kb	10kb-25kb	5kb-25kb	25kb-50kb	10kb-50kb	5kb-50kb	50kb-100kb	25kb-100kb	10kb-100kb	5kb-100kb
GRiNCH	0.726	0.738	0.713	0.739	0.704	0.699	0.728	0.719	0.683	0.657
deDoc	0.698	0.759	0.620	0.780	0.710	0.580	0.815	0.725	0.659	0.525
OnTAD	0.721	0.745	0.593	0.739	0.588	0.465	0.657	0.522	0.406	0.307
Armatus	0.686	0.526	0.427	0.756	0.465	0.408	0.755	0.688	0.457	0.383
GMAP	0.634	0.680	0.575	0.684	0.583	0.449				
matryoshka	0.581	0.635	0.616	0.664	0.608	0.501	0.592	0.576	0.551	0.512
Arrowhead	0.572	0.654	0.419	0.686	0.534	0.351	0.411	0.286	0.204	0.174
HiCKey	0.549	0.711	0.408	0.735	0.592	0.311	0.689	0.548	0.447	0.220
SpectralTAD	0.548	0.493	0.298	0.608	0.333	0.188	0.612	0.415	0.205	0.112
SuperTAD		0.781		0.859	0.721		0.789	0.727	0.602	
HiTAD	0.840				0.649	0.621				
TADpole				0.848			0.885	0.792		
TADtree							0.697			

Supplementary Table 2

Raio of Hier_SSIM between 20% and 100% versus that between 50% and 100%

	50kb		10kb	
	50%	20%	50%	20%
TADpole	0.879	0.885		
SuperTAD	0.790	0.793	0.828	0.821
deDoc	0.751	0.740	0.614	0.622
HiCKey	0.736	0.734	0.742	0.743
SpectralTAD	0.731	0.721	0.684	0.684
OnTAD	0.701	0.699	0.672	0.671
GMAP	0.652	0.706	0.721	0.697
HiTAD*			0.676	0.634
GRiNCH	0.595	0.596	0.562	0.540
TADtree	0.589	0.589		
matryoshka	0.509	0.512	0.418	0.521
Armatus	0.496	0.452	0.518	0.481

* The ratio of HiTAD equals Hier_SSIM of 20% sample to Hier_SSIM of 100% sample.

The cool inputs of 20% downsample are computed from sparse matrix by author, while those of 100% sample are cited from processed published cool files (ftp://cooler.csail.mit.edu/coolers).

Supplementary Table 3

The running time and memory cost for each of 13 methods

		100kb	50kb	25kb	10kb	5kb
Arrowhead	time	0m14.489s	0m24.141s	0m35.258s	1m6.845s	2m2.393s
	memory	2.0G	3.9G	6.7G	9.9G	10.9G
Armatus	time	0m7.490s	0m22.973s	0m53.060s	6m48.109s	32m58.254s
	memory	29.6M	116.9M	465.7M	2.8G	11.3G
TADtree	time	6m44.323s	62m27.921s	2274m51.231s		
	memory	136.7M	371.1M	499.2M		
GMAP	time		~20s	~1m	~3m	~10m
	memory		1.2G	2.2G	18.0G	27.9G
HiTAD	time	10.579s	22.462s	53.770s	5m10.099s	17m39.057s
	memory	598.8M	1.3G	1.6G	3.2G	4.4G
deDoc	time	0m22.88s	1m17.34s	4m18.36s	18m22.21s	66m0.42s
	memory	6.5G	12.3G	19.6G	26.7G	31.9G
matryoshka	time	0m7.117s	0m32.810s	2m35.674s	25m6.582s	80m33.075s
	memory	39.5M	118.3M	465.8M	2.8G	11.3G
OnTAD	time	0m0.502s	0m1.342s	0m4.492s	0m27.758s	1m59.385s
	memory	53.9M	235.1M	1.8G	11.4G	45.5G
TADpole	time	46m53.274s	113m26.482s	257m9.550s		
	memory	927.7M	2.8G	5.1G		
SpectralTAD	time	~5s	~10s	~15s	~30s	~1m
	memory	984.8M	1.2G	2.0G	6.10G	24.2G
HiCKey	time	0m0.653s	0m1.882s	0m4.289s	0m8.063s	0m11.179s
	memory	39.5M	99.0M	139.2M	275.4M	433.8M
SuperTAD	time	917m48.012s	8592m16.054s			
	memory	11.2G	88.4G			
GRiNCH	time	0m23s	0m48s	4m23s	10m22s	47m57s
	memory	522M	1750M	6326M	37154M	145508M

The cells with slash main the computational limitation in resolution coverage of method or overloaded computing memory.
