# Supplementary material for: A comprehensive benchmarking with interpretation and operational guidance for the hierarchy of topologically associating domains
Source: Nat Commun. 2024 May 23;15:4376. doi: 10.1038/s41467-024-48593-7 (PMC11116433; doi:10.1038/s41467-024-48593-7)
Supplement: Supplementary file 3 — Description of Additional Supplementary Files [file 41467_2024_48593_MOESM3_ESM.pdf]

Description of Additional Supplementary Files:

Supplementary Data 1: Genomic coverage of each caller

Supplementary Data 2: Accession numbers of dataset used
