# Supplementary material for: A comprehensive benchmarking with interpretation and operational guidance for the hierarchy of topologically associating domains
Source: Nat Commun. 2024 May 23;15:4376. doi: 10.1038/s41467-024-48593-7 (PMC11116433; doi:10.1038/s41467-024-48593-7)
Supplement: Supplementary file 6 — Reporting Summary [file 41467_2024_48593_MOESM6_ESM.pdf]

nature portfolio

Corresponding author(s): Xiaochen Bo, Jin Gu, Hebing Chen

Last updated by author(s): Apr 19, 2024

Reporting Summary

Nature Portfolio wishes to improve the reproducibility of the work that we publish. This form provides structure for consistency and transparency in reporting. For further information on Nature Portfolio policies, see our Editorial Policies and the Editorial Policy Checklist.

Statistics

For all statistical analyses, confirm that the following items are present in the figure legend, table legend, main text, or Methods section.

n/a	Confirmed	
☐	☒	The exact sample size (n) for each experimental group/condition, given as a discrete number and unit of measurement
☐	☒	A statement on whether measurements were taken from distinct samples or whether the same sample was measured repeatedly
☒	☐	The statistical test(s) used AND whether they are one- or two-sided<br>*Only common tests should be described solely by name; describe more complex techniques in the Methods section.*
☒	☐	A description of all covariates tested
☒	☐	A description of any assumptions or corrections, such as tests of normality and adjustment for multiple comparisons
☐	☒	A full description of the statistical parameters including central tendency (e.g. means) or other basic estimates (e.g. regression coefficient) AND variation (e.g. standard deviation) or associated estimates of uncertainty (e.g. confidence intervals)
☒	☐	For null hypothesis testing, the test statistic (e.g. F, t, r) with confidence intervals, effect sizes, degrees of freedom and P value noted<br>*Give P values as exact values whenever suitable.*
☒	☐	For Bayesian analysis, information on the choice of priors and Markov chain Monte Carlo settings
☒	☐	For hierarchical and complex designs, identification of the appropriate level for tests and full reporting of outcomes
☒	☐	Estimates of effect sizes (e.g. Cohen's d, Pearson's r), indicating how they were calculated

Our web collection on statistics for biologists contains articles on many of the points above.

Software and code

Policy information about availability of computer code

Data collection	SRA Toolkit v3.0.10
Data analysis	The analysis code is available at https://github.com/XiangXuCode/TAD_hierarchy_benchmark.<br>TAD hierarchy callers: Arrowhead v2.09.00, Armatus v2.3, HiTAD v0.4.2, matryoshka v1.0, OnTAD v1.2, TADpole v0.0.09000, SpectralTAD v1.2.0, GRiNCH v1.0.0, deDoc v1.0.0, SuperTAD v1.2, TADtree v1.0.0, GMAP v1.4, and HiCKey v1.0.<br>Data process: HiC-pro v2.11.4, bedGraphToBigwig v2.8, structural similarity(SSIM), deepTools v3.3.2, DESeq2 v1.26.0, cooler v0.8.11, and BEDOPS v2.4.38.<br>Base or dependences to run callers: Java v1.8.0_92, python v3.8.12 and v2.7.15(base for HiC-Pro), R v3.6.3, juicer tools v2.09.00, skiamge python package, and Ubuntu 20.04.<br>Visualization: HiCExplorer.<br>For more dependencies, please refer to README of each callers on our github space(https://github.com/XiangXuCode/TAD_hierarchy_benchmark).

For manuscripts utilizing custom algorithms or software that are central to the research but not yet described in published literature, software must be made available to editors and reviewers. We strongly encourage code deposition in a community repository (e.g. GitHub). See the Nature Portfolio guidelines for submitting code & software for further information.

Data

Policy information about availability of data

All manuscripts must include a data availability statement. This statement should provide the following information, where applicable:

- Accession codes, unique identifiers, or web links for publicly available datasets
- A description of any restrictions on data availability
- For clinical datasets or third party data, please ensure that the statement adheres to our policy

A summary of the data used in this study is shown in Supplementary Table 5. In situ Hi-C data of GM12878, GM12878 replicate sample, HMEC, HUVEC, IMR90, K562, KBM7, NHEK and CH12-LX cell lines are available in the GEO database under accession code GSE63525 [https://www.ncbi.nlm.nih.gov/geo/query/acc.cgi?acc=GSE63525]. Cool files of 5 cell types (HMEC, HUVEC, IMR90, K562, and KBM7) were downloaded from the Cooler [ftp://cooler.csail.mit.edu/coolers] before, but may be requested by email to the authors nowadays. In situ Hi-C data of drosophila S2 cell line are available in the SRA database under accession code SRR9019613 [https://trace.ncbi.nlm.nih.gov/Traces/?view=run_browser&acc=SRR9019613&display=metadata]. ChIP-seq data and RNA-seq data of GM12878 and K562 are available in the ENCODE project99 (https://www.encodeproject.org). CTCF ChIP-seq data of GM12878, K562, CH12-LX, and S2 cell are available in the ENCODE project under accession number ENCSR000DZN [https://www.encodeproject.org/experiments/ENCSR000DZN], ENCSR000DWE [https://www.encodeproject.org/experiments/ENCSR000DWE], ENCSR000ERM [https://www.encodeproject.org/experiments/ENCSR000ERM], and ENCSR711UTK [https://www.encodeproject.org/experiments/ENCSR711UTK]. For GM12878 cell line, ChIP-seq data (H3K4me3, H3K27ac, POLR2A, H3K9ac, H3K27me3, H3K9me3, and H3K4me1) are available in the ENCODE project under accession number ENCSR057BWO [https://www.encodeproject.org/experiments/ENCSR057BWO], ENCSR000AKC [https://www.encodeproject.org/experiments/ENCSR000AKC], ENCSR000EAD [https://www.encodeproject.org/experiments/ENCSR000EAD], ENCSR000AKH [https://www.encodeproject.org/experiments/ENCSR000AKH], ENCSR000AKD [https://www.encodeproject.org/experiments/ENCSR000AKD], ENCSR000AOX [https://www.encodeproject.org/experiments/ENCSR000AOX], and ENCSR000AKF [https://www.encodeproject.org/experiments/ENCSR000AKF], respectively. RNA-seq data and Dnase-seq data are available in the ENCODE project under accession number ENCSR843RJV [https://www.encodeproject.org/experiments/ENCSR843RJV], and ENCSR000EMT [https://www.encodeproject.org/experiments/ENCSR000EMT], respectively. For K562 cell line, ChIP-seq data (H3K27ac, POLR2A, H3K27me3, and H3K4me1) are available in the ENCODE project under accession number ENCSR000AKP [https://www.encodeproject.org/experiments/ENCSR000AKP], ENCSR031TFS [https://www.encodeproject.org/experiments/ENCSR031TFS], ENCSR000EWB [https://www.encodeproject.org/experiments/ENCSR000EWB], and ENCSR000EWC [https://www.encodeproject.org/experiments/ENCSR000EWC], respectively. RNA-seq data and Dnase-seq data are available in the ENCODE project under accession number ENCSR594NJP [https://www.encodeproject.org/experiments/ENCSR594NJP], and ENCSR000EKS [https://www.encodeproject.org/experiments/ENCSR000EKS], respectively. For paired colorectal and paracancerous tissues, Hi-C data and RNA-seq data of are available in the GEO databse under accession code GSE133928 [https://www.ncbi.nlm.nih.gov/geo/query/acc.cgi?acc=GSE133928], from which we downloaded the TPM count files of BRD3187 and BRD3187N. The single-cell Hi-C data of GM12878 and IMR90 are available in the 4DN project113 (https://www.4dnucleome.org/) under accession code 4DNESUE2NSGS [https://data.4dnucleome.org/experiment-set-replicates/4DNESUE2NSGS] and 4DNES4D5MWEZ [https://data.4dnucleome.org/experiment-set-replicates/4DNES4D5MWEZ].

Research involving human participants, their data, or biological material

Policy information about studies with human participants or human data. See also policy information about sex, gender (identity/presentation), and sexual orientation and race, ethnicity and racism.

Reporting on sex and gender	None.
Reporting on race, ethnicity, or other socially relevant groupings	None.
Population characteristics	None.
Recruitment	None.
Ethics oversight	None.

Note that full information on the approval of the study protocol must also be provided in the manuscript.

Field-specific reporting

Please select the one below that is the best fit for your research. If you are not sure, read the appropriate sections before making your selection.

☒ Life sciences ☐ Behavioural & social sciences ☐ Ecological, evolutionary & environmental sciences

For a reference copy of the document with all sections, see nature.com/documents/nr-reporting-summary-flat.pdf

Life sciences study design

All studies must disclose on these points even when the disclosure is negative.

Sample size	Sample size was marked in Figure 3a and Supplementary figure 4. The sample size of TAD length is determined by standard output of TAD calling methods at different resolutions.
Data exclusions	No data was excluded.
Replication	Two independent biological experience data were performed on all methods.

Randomization	Not relevant, because we did not collect biological samples in our research. The published data we used had two replications. They were all used without randomly choose.
Blinding	Not relevant, because we did not manually group data and all data processing pipelines were standardized.

Reporting for specific materials, systems and methods

We require information from authors about some types of materials, experimental systems and methods used in many studies. Here, indicate whether each material, system or method listed is relevant to your study. If you are not sure if a list item applies to your research, read the appropriate section before selecting a response.

Materials & experimental systems

n/a	Involved in the study
☒	☐ Antibodies
☒	☐ Eukaryotic cell lines
☒	☐ Palaeontology and archaeology
☒	☐ Animals and other organisms
☒	☐ Clinical data
☒	☐ Dual use research of concern
☒	☐ Plants

Methods

n/a	Involved in the study
☒	☐ ChIP-seq
☒	☐ Flow cytometry
☒	☐ MRI-based neuroimaging

Plants

Seed stocks	Not relevant.
Novel plant genotypes	Not relevant.
Authentication	Not relevant.
